# Indonesian Euphorbiaceae: Ethnobotanical Survey, In Vitro Antibacterial, Antitumour Screening and Phytochemical Analysis of *Euphorbia atoto*

**DOI:** 10.3390/plants12223836

**Published:** 2023-11-13

**Authors:** Dyke Gita Wirasisya, Annamária Kincses, Lívia Vidács, Nikoletta Szemerédi, Gabriella Spengler, Anita Barta, I Gde Mertha, Judit Hohmann

**Affiliations:** 1 Institute of Pharmacognosy, University of Szeged, H-6720 Szeged, Hungary; dykegita_w@unram.ac.id (D.G.W.); kincses.annamaria@szte.hu (A.K.); vidacs.livia@gmail.com (L.V.); bartaanita96@gmail.com (A.B.); 2 Department of Pharmacy, Faculty of Medicine, University of Mataram, Mataram 83126, Indonesia; 3 Department of Medical Microbiology, Albert Szent-Györgyi Health Center and Albert Szent-Györgyi Medical School, University of Szeged, H-6725 Szeged, Hungary; szemeredi.nikoletta@med.u-szeged.hu (N.S.); spengler.gabriella@med.u-szeged.hu (G.S.); 4 Department of Biology Education, Faculty of Teacher Training and Education, University of Mataram, Mataram 83126, Indonesia; gdemerth19@gmail.com; 5 ELKH-USZ Biologically Active Natural Products Research Group, University of Szeged, H-6720 Szeged, Hungary

**Keywords:** traditional medicine, Euphorbiaceae, antimicrobial assay, antitumour assay, *Euphorbia atoto*

## Abstract

Indonesia is among the countries with the most significant biodiversity globally. *Jamu*, the traditional medicine of Indonesia, predominantly uses herbal materials and is an integral component of the Indonesian healthcare system. The present study reviewed the ethnobotanical data of seven Indonesian Euphorbiaceae species, namely *Euphorbia atoto*, *E. hypericifolia*, *Homalanthus giganteus*, *Macaranga tanarius*, *Mallotus mollissimus*, *M. rufidulus*, and *Shirakiopsis indica*, based on the RISTOJA database and other literature sources. An antimicrobial screening of the plant extracts was performed in 15 microorganisms using the disk diffusion and broth microdilution methods, and the antiproliferative effects were examined in drug-sensitive Colo 205 and resistant Colo 320 cells by the MTT assay. The antimicrobial testing showed a high potency of *M. tanarius*, *H. giganteus*, *M. rufidulus*, *S. indica*, and *E. atoto* extracts (MIC = 12.5–500 µg/mL) against different bacteria. In the antitumour screening, remarkable activities (IC_50_ 0.23–2.60 µg/mL) were demonstrated for the extracts of *H. giganteus*, *M. rufidulus*, *S. indica*, and *E. atoto* against Colo 205 cells. The *n*-hexane extract of *E. atoto*, with an IC_50_ value of 0.24 ± 0.06 µg/mL (Colo 205), was subjected to multistep chromatographic separation, and 24-methylene-cycloartan-3*β*-ol, jolkinolide E, tetra-*tert*-butyl-diphenyl ether, *α*-tocopherol, and *β*-sitosterol were isolated.

## 1. Introduction

Indonesia is considered to have substantial biodiversity based on its high number of indigenous plants. From the 40,000 tropical plant species that are native to Indonesia, 39% are considered endemics. Most Indonesians, especially those in rural regions, employ traditional herbal medicines, collectively known as jamu, to treat diseases because of the abundance of medicinal plants. Besides their medicinal uses, certain plants are used for specific purposes, like tribal ceremonies, whereas others are grown for daily consumption and trade [1,2].

The Health Ministry of the Republic of Indonesia, through the National Institute of Health Research and Development (NIHRD) in collaboration with some leading universities, has conducted comprehensive research on medicinal plants and jamu (“Riset Tanaman Obat dan Jamu”, RISTOJA). RISTOJA is an ethnomedicinal research program that explores the utilization of medicinal plants in Indonesia. The RISTOJA database collected findings for 32,014 medicinal plants in Indonesia during 2012–2017 from 405 ethnic groups in 34 provinces. Since access to the RISTOJA database is not open to the public, data can only be used through proposals submitted to the NIHRD. The Indonesian government, industry, and academic communities agree that further studies are needed to determine the efficacy and safety of different traditional jamu preparations to ensure their safe and effective use.

The analysis of jamu preparations as rational phytotherapies requires a variety of pharmacological and phytochemical studies of extracts and isolated compounds. The results of in vitro and in vivo preclinical studies can support or refute the traditional use of medicinal plants. Scientific investigations of medicinal plants used in indigenous medical systems have been increasingly relevant, and the role of ethnobotanical information in the search for new drugs has been of continuous importance. Knowledge of traditional medicine can serve as the starting point for drug discovery. Therefore, screening of the biological activity of the used plants can provide valuable data for selecting plant extracts for isolating and identifying the active ingredients [3].

Continuing our current research interest in species of the spurge family [4,5], the present study (a) reviewed the data on the traditional therapeutic uses of seven Euphorbiaceae species, namely *Euphorbia atoto* G.Forst., *E. hypericifolia* L., *Homalanthus giganteus* Zoll. & Moritzi, *Macaranga tanarius* Müll.Arg., *Mallotus mollissimus* (Geiseler) Airy Shaw, *M. rufidulus* Müll.Arg., and *Shirakiopsis indica* (Willd.) Esser; (b) conducted an in vitro antimicrobial and antitumour screening of extracts with different polarities prepared from the plants; and (c) isolated compounds from *E. atoto*. This study used the metadata from the RISTOJA collected in 2012, 2015, and 2017 together with ethnobotanical data from the literature to evaluate the traditional uses of the seven selected species. The antimicrobial activities of the extracts were investigated in seven Gram-positive, four Gram-negative, and four fungal strains, and their antiproliferative activity was examined in human colon adenocarcinoma cell lines (Colo 205 and Colo 320). The *n*-hexane extract of *E. atoto* that exhibited a strong antiproliferative effect was subjected to multistep chromatographic separation to isolate five compounds (**1**–**5**), among which, four were isolated for the first time from this species.

## 2. Results and Discussion

### 2.1. Ethnobotanical Data

#### 2.1.1. Illness Categories

Fifty-six ethnobotanical uses were described in the RISTOJA database and in the literature for the selected seven Euphorbiaceae species (*E. atoto*, *E. hypericifolia*, *H. giganteus*, *M. tanarius*, *M. mollissimus*, *M. rufidulus*, and *S. indica*) and classified into 12 categories (Table 1). The categorization was based on the ICPC-3 (available at https://www.who.int/standards/classifications/other-classifications/international-classification-of-primary-care, accessed on 15 June 2023) that employs the perception of patients or interviewees to classify ethnobotanical information. This framework aligns with the objective of ethnobotanical research for studying the indigenous aspects of medicinal plants [6].

Using the categorization of the ICPC-3, the majority of plants were frequently utilized to treat skin-related problems (17). Other relevant uses were associated with general symptoms (10), digestive system ailments (9), and musculoskeletal issues (6). The plants were less frequently employed to address genital system concerns (3), pregnancy and childbearing issues (3), eye-related conditions (2), neurological system disorders (2), respiratory system conditions (1), blood-related disorders and immune system conditions (1), endocrine, metabolic, and nutritional system disorders (1), and urinary system ailments (1).

#### 2.1.2. Ethnobotanical Uses of the Selected Species

In our study, the most frequently cited plant species were *M. tanarius* and *E. atoto*, accounting for 78% of the reported uses (Table 1). *M. tanarius* is a tree commonly found in wet tropical regions globally, including all islands of Indonesia [7]. It has a long history of traditional medicinal use, particularly in the Pacific region [8,9,10,11,12]. *M. tanarius* was mentioned 35 times in the RISTOJA database and other literature, and it has been used to treat various ailments. It is most notable for its activity against skin-related problems such as sores, boils, and itching and for its wound-healing effects (Table 2). This plant is frequently applied for the treatment of gastrointestinal disorders, such as a gastric ulcer, stomach ache, and diarrhoea. Infectious diseases, such as malaria, eye diseases, bloody diarrhoea, and sores, were also listed among its traditional applications. The pharmacological activities of the plant have been studied by several groups, and antidiabetic, antiacetylcholinesterase, cytotoxic, and antiherbivore activities were reported [13,14,15,16,17]. These activities can be attributed to the presence of numerous compounds, specifically prenylated flavonoids that have been isolated from this species [17,18,19,20,21,22].

*E. atoto* is a shrub primarily found in wet, sandy, tropical biomes, particularly in sandy beach areas or dunes near the seashore [23]. *E. atoto* was mentioned nine times in the RISTOJA database and other literature, and it was mainly used to treat skin-related conditions. The leaf, bark, and whole plant were used in Jambi, Indonesia, the Nicobar Islands of India, and Samoa to treat ulcers, sores, old wounds, abscesses, rheumatism, lumbago, and headaches (Table 2). Despite the potential therapeutic applications of this species, there have been limited pharmacological investigations of its effects. Trachylobane (euphoratones A–B, 3-oxo-*ent*-trachyloban-17-oic acid), *ent*-kaurane (*ent*-kauran-16*β*-ol-3-one, *ent*-kaurane-3-oxo-16*β*,17-diol, *ent*-kaurane-3*β*,16 *β*-diol), *ent*-sanguinolane diterpenoids, baccatin, campesterol, stigmasterol, *β*-sitosterol, and (+)-9-aza-1-methylbicyclo [3,3,1]nonan-3-one were identified in *E. atoto* in previous studies [24,25,26]. Some of the isolated diterpenoids were found to have weak anti-inflammatory activity against mouse RAW264.7 cells [26]. It is important to note that the research conducted by Norhanom and Yadav in 1995 described the tumour-promoting activity of several Malaysian plants belonging to the Euphorbiaceae family, including *Euphorbia atoto*, which was tested in vitro on a human lymphoblastoid cell line harbouring the Epstein-Barr virus (EBV) genome. Based on this observation, the authors pointed out that regular use of *E. atoto* could well be an etiological factor for the promotion of tumours among rural Malaysian peoples [27].

Regarding *E. hypericifolia*, its traditional uses in South Africa and Uganda included the treatment of gonorrhoea, snakebites, and scorpion bites. Previous pharmacological studies demonstrated its anticancer effects against colorectal cancer [28].

*S. indica* fruits and leaves were utilized to treat constipation and measles, respectively, in the traditional medicine of Thailand and North Sumatra. *S. indica* has been studied for its anti-*Helicobacter pylori*, anti-HIV, wound-healing, and anti-inflammatory properties [29,30,31,32].

*H. giganteus*, *M. rufidulus*, and *M. mollissimus* were used in the healing system of Indonesia, and their indications included urinary tract diseases, bone injury, fever, and flatulence (Table 2). No records were found regarding pharmacological investigations of these plants.

**Table 2:** Ethnobotanical data related to the selected species.

Species	Part Used	Location	Traditional Uses	References
*M. tanarius* (L.) Müll.Arg.	Leaf	Aceh	High cholesterol	[8]
		West Java	Itchy, skin diseases in babies, wounds, gastric ulcers	[8]
		Gorontalo	Itchy	[8]
		North Sulawesi	Leucorrhoea, diarrhoea, sores, wounds, stomach ache	[8]
		Banten	Gastric ulcer	[33]
		Southeast Sulawesi	Internal organ treatments	[11]
		West Timor	Malaria	[8]
		North Maluku	Fertility, eye diseases	[8,11]
		North Sumatera	Muscle injury	[10]
	Bark	Papua	Eye diseases, skin diseases, malaria, headache, snake bite treatment, lymphoid problems	[11]
		Maluku	Bloody diarrhoea, postpartum care, mouth ulcer	[8,9]
		West Java	Haemorrhoids	[8]
		East Nusa Tenggara	Bone injury, postpartum haemorrhage	[11]
	Fruits	Papua	Malaria	[11]
	Root	North Maluku	Cough	[10]
	Exudate/sap	Southeast Maluku	Boils	[11]
		North Maluku	Skin diseases	[10]
		Queensland	Sores, wound healing	[12]
*M. mollissimus* (Geiseler) Airy Shaw.	Leaf	Eastern Indonesia *	Urinary tract diseases	[34]
		East Nusa Tenggara	Flatulence	[11]
	Bark	East Nusa Tenggara	Bone injury	[11]
*H. giganteus* Zoll. & Moritzi	Leaf	North Sumatra	Itchy, fever	[35]
	Seeds		Fever	[36]
*M. rufidulus* (Miq.) Müll.Arg.	Leaf	West Kalimantan	Bone injury	[37]

*S. indica* (Willd.) Esser.	Fruits	Thailand	Constipation	[38]
	Leaf	North Sumatra	Measles	[39]
*E. atoto* G.Forst.	Leaf	Jambi	Postpartum care	[11]
		Nicobar Islands	Ulcer, sores, old wound healing	[40]
			Rheumatism, lumbago	[40]
	Bark	Jambi	Headache	[11]
	Whole plants	Samoa	Wound healing, abscess	[41]
*E. hypericifolia* L.	Leaf	South Africa	Gonorrhoea	[42]
	Juice	Uganda	Snake bite treatment, scorpion bite treatment	[43]

* Eastern Indonesia includes the islands of Bali, Nusa Tenggara, Sulawesi, Maluku and Papua.

#### 2.1.3. Plant Parts Used

**Figure 1:**
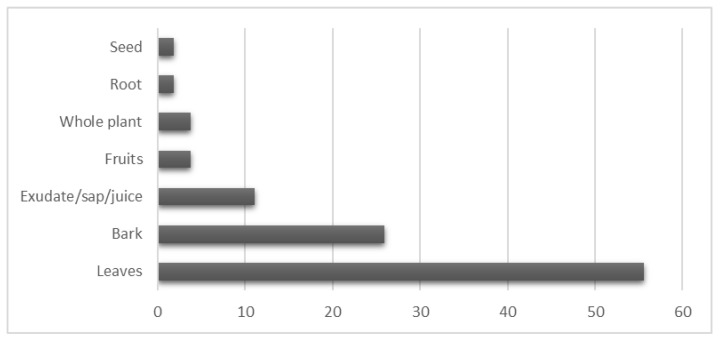
Frequencies of the use of medical plants parts (%).

From the collected ethnobotanical data, more than 56% of the recorded symptoms were treated using leaves. Leaves were commonly used in the treatment of skin-related problems (*M. tanarius*, *H. giganteus*, *S. indica*, and *E. atoto*) as well as digestive system disorders (*M. tanarius* and *M. mollissimus*). The second most frequently used plant part was the bark, accounting for more than 25% of the total utilization. Exudates/sap/juices constituted 11% of the mentioned remedies, whereas whole plants and fruits each represented 4% of the applications. The least utilized plant parts were seeds and roots, accounting for 2% each (Figure 1). The predominant use of leaves as the primary plant part in traditional medicine is well documented by numerous studies [44,45,46]. This preference for leaves can be attributed to their abundance and easy accessibility in nature. Leaves offer versatility in their application, as they can be utilized in dried, fresh, or processed forms. Additionally, leaves are known for their convenience in preservation, further contributing to their widespread usage in traditional medicinal practices [36].

### 2.2. Antimicrobial Activity Screening

The antimicrobial activities of the extracts of *E. atoto*, *E. hypericifolia*, *H. giganteus*, *M. tanarius*, *M. mollissimus*, *M. rufidulus*, and *S. indica* were tested against 15 microorganisms, including Gram-positive (seven), Gram-negative (four), and fungal (four) strains. The dried plant materials were extracted with MeOH, and after evaporation, the extracts were subjected to a solvent–solvent partition to obtain *n*-hexane, chloroform, ethyl acetate, and aqueous methanolic extracts. All 28 extracts of different polarities were first screened for antimicrobial activity by the disk diffusion method, and extracts exhibiting high activities were tested by the broth microdilution method. This is the first comprehensive analysis of the antimicrobial effects of extracts of these Euphorbiaceae species.

#### 2.2.1. Screening by the Disk Diffusion Method

The screening results illustrated that the strains most sensitive to the tested extracts were *S. aureus* ATCC 29213, *M. catarrhalis* ATCC 25238, and *N. glabrata* ATCC 2001 (Table S1). In general, better activities were recorded against Gram-positive bacteria than against Gram-negative bacteria, excluding *M. catarrhalis* ATCC 25238, against which remarkable activities were detected. Among the fungal strains, noteworthy inhibitory activities were registered against *C. parapsilosis* ATCC 22019 and *N. glabrata* ATCC 2001. The largest zones of inhibition were measured for *N. glabrata* ATCC 2001 (up to 33 mm).

The *M. tanarius* extracts were most effective against Gram-positive strains, and its ethyl acetate extract had zones of inhibition larger than 15 mm against six bacterial and fungal strains. The *M. mollissimus* and *M. rufidulus* extracts exhibited modest activities against Gram-positive bacteria, but they were more effective against the *N. glabrata* ATCC 2001 stain. Among the *H. giganteus* extracts, the ethyl acetate was most potent against *Staphylococcus* strains, *M. catarrhalis* ATCC 25238, *C. parapsilosis* ATCC 22019, and *N. glabrata* ATCC 2001. In the case of the *S. indica* extracts, the antifungal effects predominated in addition to effects against *M. catarrhalis* ATCC 25238. Extracts of the two *Euphorbia* species displayed similar antimicrobial effects with moderate potency against bacteria and higher activity against fungal strains (Table S1).

#### 2.2.2. Determination of MICs by the Microdilution Method

Seventeen extracts displaying significant activity (>15 mm diameter) in the antimicrobial screening by the disk diffusion method were further investigated, and their MICs were determined by the broth microdilution method (Table 3). The strongest activities were measured for the *M. tanarius*, *H. giganteus*, and *E. atoto* ethyl acetate extracts against *M. catarrhalis* ATCC 25238 (MIC = 12.5 µg/mL), followed by the *S. indica* aqueous methanolic extract (MIC = 25 µg/mL). Outstanding effects of the *M. tanarius* chloroform extract (MIC = 15.6 µg/mL) and *H. giganteus* ethyl acetate extract (MIC = 25 µg/mL) were observed against *S. epidermidis* ATCC 12228. The chloroform extract of *M. rufidulus* was the only extract in our study with potency against *B. subtilis* ATCC 6633 (MIC = 25 µg/mL). The *M. tanarius* ethyl acetate extract was the most effective against *N. glabrata* ATCC 2001 (MIC = 50 µg/mL). Further moderate activities (MIC = 100–500 µg/mL) were recorded against *S. aureus* ATCC 29213, MRSA ATCC 43300, *C. parapsilosis* ATCC 22019, and *N. glabrata* ATCC 2001. None of the *n*-hexane extracts was effective as an antimicrobial agent, but it is noteworthy that the ethyl acetate extract exhibited more favourable results in comparison to other solvents. The advantageous use of ethyl acetate as an extraction solvent has been mentioned in previous publications [47,48].

### 2.3. Antitumour Activity

Extracts with different polarities prepared from *E. atoto*, *E. hypericifolia*, *H. giganteus*, *M. tanarius*, *M. mollissimus*, *M. rufidulus*, and *S. indica* were tested for antiproliferative activity in doxorubicin-sensitive Colo 205 cells and doxorubicin-resistant Colo 320/MDR-LRP cells using the MTT assay. The aim of this experiment was to map the antitumour potential of the plant extracts because antiproliferative, cytotoxic, and multidrug resistance-reversing activities are characteristic of many Euphorbiaceae species [49].

Strong antiproliferative activity (IC_50_ < 1 µg/mL) was observed for the *n*-hexane and chloroform extracts of *E. atoto* and *H. giganteus* and the chloroform extracts of *M. rufidulus* and *S. indica* (IC_50_ = 1.04 µg/mL) in Colo 205 cells (Table 4). Colo 320 cells were less sensitive, and the highest activities, as indicated by IC_50_ values, ranged from 7 to 12 µg/mL. In most cases, the antiproliferative activity of the extracts was stronger against Colo 205 cells than against Colo 320 cells. The chloroform extract of *M. tanarius* was an exception because it had IC_50_ values 8.46 ± 0.36 µg/mL and 23.02 ± 0.86 µg/mL for Colo 320 and Colo 205 cells, respectively. This indicates that the *M. tanarius* chloroform extract has a selective antiproliferative effect in drug-resistant cells, and a relative resistance ratio (RR) of 0.37 was calculated as the ratio of the IC_50_ between the resistant and sensitive cancer cell lines. RR ≤ 0.5 indicates that an extract contains compounds with drug resistance-modifying activity and possesses collateral sensitivity effects [50]. Extracts of *E. hypericifolia* were marginally effective as an antiproliferative agent (*n*-hexane extract: IC_50_ = 55.94 ± 0.64 µg/mL; chloroform extract: IC_50_ = 36.54 ± 0.64 µg/mL) against Colo 205 and Colo 320 cells; however, its compounds displayed cytotoxic activities against the colorectal cancer cell line HCT-116 [28].

Interestingly, in this screening, none of the ethyl acetate and aqueous methanolic extracts exhibited antiproliferative activity (Table 4).

### 2.4. Isolation and Identification of Compounds from E. atoto

**Figure 2:**
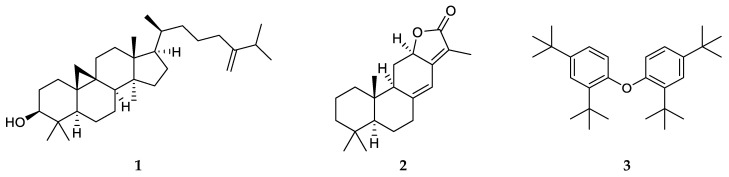
Structures of compounds 1–3 isolated from *E. atoto*.

Among the studied Euphorbiaceae species, *E. atoto* was selected for detailed phytochemical studies because its *n*-hexane extract displayed strong antitumour activity (IC_50_ = 0.24 ± 0.06 µg/mL), and it was available in an appropriate amount (8.12 g). The *n*-hexane extract was first separated using OCC on polyamide, and the fractions obtained in this chromatography were then purified using VLC, PTLC, HPLC, and crystallization to yield five compounds (**1**–**5**, Figure 2). The structures of the isolated compounds were determined using ^1^H NMR, ^13^C NMR JMOD, ^1^H–^1^H COSY, HSQC, HMBC, and NOESY experiments.

Compound **1** was identified as 24-methylene-cycloartan-3*β*-ol based on a comparison of its spectral data with literature data [51]. This compound was previously isolated from several Euphorbia species [52]. Compound **2** was revealed to be identical to jolkinolide E based on its NMR spectroscopic characteristics. Jolkinolide E (**2**) is an abietane diterpene previously found in *E. jolkini*, *E. helioscopia*, and *E. calyptrate* [53].

Compound **3** was identified as tetra-*tert*-butyl-diphenyl ether [1,10-oxybis(2,4-di-tert-butylbenzene)], and it is an unusual structural type regarding the presence of *tert*-butyl groups in the molecule. Despite its “unnatural” structure, it was previously isolated from nature twice. Specifically, tetra-*tert*-butyl-diphenyl ether (**3**) was obtained from the marine mollusc *Onchidium struma* [54] and *Dioscorea collettii* rhizomes [55]. Jing et al. supposed that this unique structure is most likely attributed to the environmental stresses of plants (e.g., salinity, water supply). The monomer of **3**, 2,4-di-*tert*-butylphenol (2,4-DTBP), has been detected in different living organisms, such as bacteria, fungi, bryophytes, pteridophytes, and higher plants. It was presumed that endocidal regulation is the primary function of 2,4-DTBP derivatives in the producing organisms [56].

Two widely occurring compounds were also isolated from the *n*-hexane extract of *E. atoto*: α-tocopherol (**4**) [57] and *β*-sitosterol (**5**) [58].

### 2.5. Investigation of the Antitumour Effects of ***1***

Among the isolated compounds, 24-methylene-cycloartan-3*β*-ol (1) was available in the amount required for cytotoxic assays. The antiproliferative activity of the compound was tested in colon adenocarcinoma cells (Colo 205), breast cancer cells (HTB-26, MCF-7), and nontumourous, human foetal lung fibroblasts (MRC-5) by the MTT assay. Compound **1** exhibited antiproliferative effects against the HTB-26 and Colo 205 cells with an IC_50_ of 24.83 µM and 30.68 µM, respectively (Table 5). Compound **1** had similar activity as the positive control, cisplatin (IC_50_ = 26.64 µM), but it displayed less tumour cell selectivity, as it was more cytotoxic in the MRC-5 cells (IC_50_ = 15.67 µM).

## 3. Materials and Methods

### 3.1. Plant Materials

**Figure 3:**
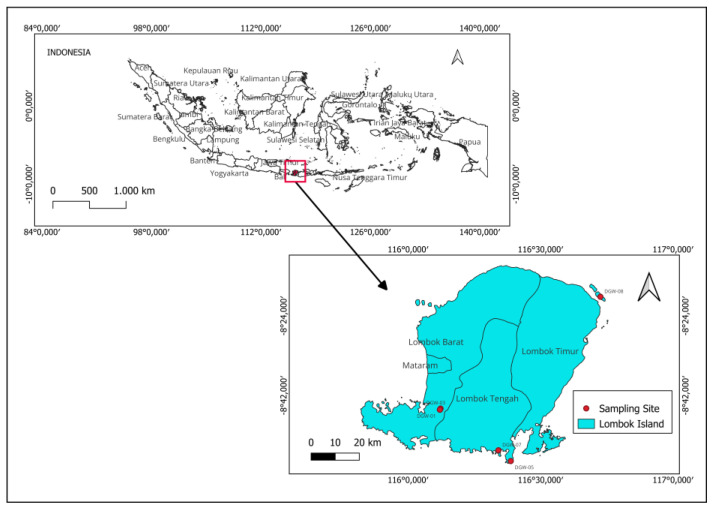
Map of Indonesia and the areas from which plants were collected.

Plant materials were collected in August 2021 from Lombok, West Nusa Tenggara, Indonesia (Figure 3). The identification of the plants was performed by I Gde Mertha (Department of Biology Education, Faculty of Teacher Training and Education, University of Mataram, Mataram, Indonesia). Voucher specimens were prepared for each species and deposited in the Laboratory of Silviculture, Department of Forestry, Faculty of Agriculture, University of Mataram (Table 6). The scientific names of the plants cited were verified in accordance with The World Flora Online [59]. The collected plant materials were washed under running tap water, cut into appropriate sizes, and airdried in an oven (Memmert, Universal Oven UN 55, Schwabach, Germany) at 40 °C ± 2 °C. 

### 3.2. Ethnobotanical Data and Analysis

The primary ethnopharmacological data of the plants was extracted from the RISTOJA collected in 2012, 2015, and 2017. This database is not accessible to the public, and permission was requested from the NIHRD, Ministry of Health of the Republic of Indonesia to use the data for seven Euphorbiaceae species in this study. One of the authors (D. G. Wirasisya) participated in the collection of data in the RISTOJA program in 2017. Additional ethnobotanical information was obtained from the following databases: Web of Knowledge, Science Direct, PubMed, SciFinder, and Google Scholar. Initially, the ethnobotanical information obtained from the RISTOJA was documented in the local language. A careful process was undertaken to translate the data into English medical terminology. Then, all of the information was combined and categorized according to the classification framework of the International Classification of Primary Care (ICPC) system developed by the Wonca International Classification Committee and accepted by the World Health Organization.

### 3.3. General Experiment Procedures

NMR spectra were recorded in CDCl_3_ or CD_3_OD on a Bruker Avance DRX 500 spectrometer (Bruker, Billerica, MA, USA) at 500 (^1^H) or 125 MHz (^13^C). The signals from the deuterated solvents were taken as references. Two-dimensional NMR measurements were obtained using the standard Bruker software. In the ^1^H–^1^H COSY, NOESY, HSQC, and HMBC experiments, gradient-enhanced versions were applied. The separation by open column chromatography (OCC) was performed using polyamide (MP Polyamide, 50–160 μm, MP Biomedicals, Irvine, CA, USA), normal-phase vacuum liquid chromatography (NP-VLC) was conducted using silica gel (15 μm, Merck, Darmstadt, Germany). Thin-layer chromatography (TLC) was performed on silica 60 F_254_ (Merck) with detection under UV light (254 and 336 nm). Concentrated sulfuric acid was used as the spray reagent, followed by heating at 105 °C for 5 min. High-performance liquid chromatography (HPLC) purification was performed on the Shimadzu LC-10AS, equipped with an SPD-10A UV–VIS detector (Shimadzu Inc., Kyoto, Japan) using the LiChrosphere Si 60 column (5 µm, 250 × 4 mm, Merck).

### 3.4. Preparation of Plant Extracts and Fractions

The dried plant materials were ground into a powder using a mechanical blender (Retsch, Grindomix GM 200, Hann, Germany) and percolated with MeOH at room temperature. The crude extracts were then concentrated in vacuo. The concentrated extracts were dissolved in 50% aqueous methanol and subjected to a continuous solvent–solvent partition to afford *n*-hexane, chloroform, ethyl acetate, and water-soluble residual fractions. The yields of the extracts and fractions are presented as percentages of the total mass of the dried plant material (Table 7).

### 3.5. Isolation of Compounds from E. atoto

The *n*-hexane fraction (8.12 g) obtained from *E. atoto* (Table 7) was subjected to open column chromatography (OCC) on polyamide using mixtures of MeOH–H_2_O (4:6, 6:4, 8:2, and 100% MeOH) as eluents. The fractions yielded from 40% and 60% (0.63 g and 0.32 g, respectively). The MeOH elutions by polyamide OCC were found to be free of chlorophyll. These fractions were combined and further separated using VLC on silica gel. The separation was performed using a gradient system of cyclohexane–ethyl acetate–EtOH (100:0:0, 90:10:0, 80:20:0, 70:30:0, 60:40:1, 50:50:1, 50:50:5, 50:50:10, 0:80:20). TLC monitoring was employed, and fractions with similar composition were combined, resulting in 17 fractions (A–Q). Compound **1** (17 mg) was obtained by crystallization from the MeOH solution of fraction B (48.7 mg). The remaining mother liquor of fraction B was subjected to further separation via normal phase (NP)-HPLC using a mobile phase of cyclohexane–ethyl acetate (94:6) that led to the isolation of compound **2** (0.7 mg). 

The fraction obtained by polyamide OCC using an elution with 80% MeOH (4.36 g) was separated using VLC on silica gel utilizing the gradient system of cyclohexane–ethyl acetate–EtOH (100:0:0, 90:10:0, 80:20:0, 70:30:0, 60:40:1, 50:50:1, 50:50:5, 50:50:10, 0:80:20), yielding 15 fractions (A–O). Fraction B (305.9 mg) was further purified by VLC on silica gel by employing a gradient system of *n*-hexane–chloroform–acetone (80:10:0, 60:40:0, 60:40:0.5, 80:20:0.5, 60:40:10, and 60:40:5), resulting in the isolation of ten subfractions (B1–10). Subfraction B8 (219.6 mg) was subsequently subjected to preparative TLC (PTLC) using cyclohexane–chloroform–acetone (6:4:0.3) as the mobile phase, yielding five subfractions (B8/1–5). PTLC using a mobile phase of cyclohexane–ethyl acetate (9:1) was then applied to separate B8/4 (115.9 mg), resulting in five subfractions (B8/4/1–5). The final purification of subfraction B8/4/3 (41.9 mg) was accomplished using NP-HPLC with cyclohexane–ethyl acetate (94:6) as the mobile phase, affording pure compounds **3** (0.7 mg) and **4** (2.8 mg). Compound **5** (60.4 mg) was obtained using crystallization from the MeOH solution of fraction E (437.5 mg).

### 3.6. Bacterial and Fungal Strains and Culture Conditions for Antimicrobial Assays

The test microorganisms included the standard Gram-positive strains *Staphylococcus aureus* (ATCC 29213), methicillin-resistant *S. aureus* (MRSA) (ATCC 43300), *S. epidermidis* (ATCC 12228), *Streptococcus pyogenes* (ATCC 19615), *S. agalactiae* (ATCC 13813), *Bacillus subtilis* (ATCC 6633), and *Enterococcus faecalis* (ATCC 29212). The standard Gram-negative strains were *Escherichia coli* (ATCC 35218), *E. coli* (K-12 AG-100), *Pseudomonas aeruginosa* (ATCC 27853), and *Moraxella catarrhalis* (ATCC 25238). The fungal strains used were *Candida albicans* (ATCC 10231), *C. parapsilosis* (ATCC 22019), *C. tropicalis* (ATCC 750), and *Nakaseomyces glabrata* (ATCC 2001). The bacterial cultures were grown on standard Mueller–Hinton (MH) agar, and fungal cultures were grown on RPMI plates (Diagnosticum Zrt., Budapest, Hungary) at 37 °C overnight in an aerobic environment.

### 3.7. Determination of Antibacterial Activity Using the Disk Diffusion Method

The disk diffusion method was employed to screen extracts for their antibacterial and antifungal activities against standard bacterial and fungal strains to determine their zones of inhibition. Briefly, the samples were dissolved in DMSO at a concentration of 50 mg/mL. Sterile filter paper disks [6 mm in diameter, Whatman antibiotic paper disk (Cytiva, Marlborough, MA, USA)] coated with 10 μL of the sample solutions were placed on top of the bacterial suspension (inoculum: 0.5 McFarland, 1.5 × 10^8^ CFU mL^−1^). Disks containing antibiotics (ciprofloxacin and ampicillin) and antifungal agents (nystatin) were used as positive controls, and disks containing DMSO served as the negative controls. Under aerobic conditions, the plates were incubated at 37 °C ± 2 °C for 20 h. The diameters of the zones of inhibition caused by the compounds, including the disk, were measured in triplicate. For each of the three repetitions, an average zone of inhibition was calculated [60].

### 3.8. Determination of the Minimum Inhibitory Concentration (MIC) 

Bacteria: The MICs of all tested fractions were determined according to the Clinical and Laboratory Standards Institute (CLSI) guidelines in three independent assays [61]. The starting concentration of tested fractions was 200 µg/mL or 500 µg/mL.

Fungi: Determination of the MIC values was performed according to the European Committee on Antimicrobial Susceptibility Testing (EUCAST) standards in three independent assays [62]. The starting concentration of tested fractions was 200 µg/mL or 500 µg/mL.

The values are given as the mean determined for three replicates from three independent experiments. DMSO was assayed to ensure there was no antibacterial and antifungal effect at the concentration (2 *v*/*v*%) applied in the assay.

### 3.9. Cell Line Cultures

Two human colon adenocarcinoma cell lines, namely Colo 205 (ATCC-CCL-222) doxorubicin-sensitive parent cells and Colo 320/MDR-LRP (ATCC-CCL-220.1) doxorubicin-resistant cells expressing ABCB1, were purchased from LGC Promochem (Teddington, UK). The cells were cultured in RPMI 1640 medium supplemented with 10% heat-inactivated foetal bovine serum (FBS), 2 mM L-glutamine, 1 mM sodium pyruvate, and 100 mM HEPES. HTB-26 breast adenocarcinoma cell line was purchased from LGC Promochem (Teddington, UK). The cell line was cultured in RPMI 1640 medium supplemented with 10% heat-inactivated foetal bovine serum, 2 mM L-glutamine, and 1 mM sodium pyruvate. The breast cancer cell line, MCF-7 (ATCC HTB-22), was purchased from LGC Promochem (Teddington, UK). The MCF-7 cell line was grown in Eagle’s minimal essential medium (EMEM), containing 4.5 g/L glucose supplemented with a non-essential amino acid mixture, a selection of vitamins, and 10% heat-inactivated FBS. The MRC-5 human embryonic lung fibroblast cell line (ATCC CCL-171) was purchased from LGC Promochem (Teddington, UK). The cell line was cultured in EMEM supplemented with a non-essential amino acid mixture, a selection of vitamins, 10% FBS, 2 mM L-glutamine, 1 mM Na-pyruvate, nystatin, and a penicillin–streptomycin mixture in concentrations of 100 U/L and 10 mg/L, respectively. All of the cells were incubated at 37 °C in a 5% CO_2_ and 95% air atmosphere.

### 3.10. Antiproliferative Assay

The antiproliferative effects of the extracts were tested in decreasing serial dilutions (2-fold dilutions starting from 100 µg/mL) in human cancer cell lines (Colo 205, Colo 320) in 96-well, flat-bottomed microtiter plates. First, the extracts were diluted in 100 µL of the medium, and then 6 × 10^3^ cells (Colo 205, Colo 320) in 100 µL of RPMI medium were added to each well, excluding the medium control wells. The culture plates were incubated at 37 °C for 72 h. Following incubation, 20 μL of 3-(4,5-dimethylthiazol-2-yl)-2,5-diphenyl tetrazolium bromide (MTT) solution (from a 5 mg/mL stock solution) was added to each well. After incubation at 37 °C for 4 h, 100 μL of the sodium dodecyl sulphate (SDS) solution (10% SDS in 0.01 M HCl) was added to each well, and the plates were further incubated at 37 °C overnight. Cell growth was determined by measuring the optical density at 540 nm (ref.: 630 nm) using a Multiskan EX ELISA reader (Thermo Labsystems, Cheshire, WA, USA). The concentration that decreased cell viability by 50% was expressed as the IC_50_ (µg/mL) for each extract. The antiproliferative effect of compound **1** was determined on the Colo 205, HTB-26, MCF-7, and MRC-5 as described for the extracts. Meanwhile, a 5 mM stock solution in DMSO and a starting concentration of 100 µM were applied. 

## 4. Conclusions

Ethnobotanical data were collected for seven Indonesian Euphorbiaceae species from the RISTOJA database and other literature sources and evaluated from different perspectives. It was stated that *E. atoto*, *E. hypericifolia*, *H. giganteus*, *M. tanarius*, *M. mollissimus*, *M. rufidulus*, and *S. indica* are medicinal plants of the jamu healing system utilized in Indonesia and surrounding areas. *E. hypericifolia* was the only species with no record in Asian ethnomedicine. However, this plant is used in South Africa and Uganda to treat gonorrhoea and snake and scorpion bite. The ethnobotanical uses of the seven plants included the treatment of skin disorders (17), general symptoms (10), digestive system ailments (9), and musculoskeletal issues (6). Many plant utilizations, such as abscesses, sores, boils, and other skin diseases; fever; urinary tract problems; cough; bloody diarrhoea; leukorrhea; and eye diseases, could be related to bacterial or fungal infections, whereas other symptoms could be reflective of colon cancer, such constipation, flatulence, stomach aches, and diarrhoea. Therefore, the plant extracts were screened for antimicrobial and antitumour activities. Fractions of different polarities (*n*-hexane, chloroform, ethyl acetate, and aqueous methanolic) were prepared from the plants and tested against 15 microorganisms, including Gram-positive (seven), Gram-negative (four), and fungal (four) strains. Antibacterial effects were observed for the *M. tanarius*, *H. giganteus*, and *M. rufidulus* extracts against the Gram-positive strains, *S. aureus* ATCC 29213, MRSA ATCC 43300, *S. epidermidis* ATCC 12228, and *B. subtilis* ATCC 6633 (MIC = 15.6–500 µg/mL). The Gram-negative bacteria, *E. coli* ATCC 35218, *E. coli* K-12 AG-100, and *P. aeruginosa* ATCC 27853, were not sensitive to most plant extracts, whereas strong antibacterial activity was observed for the *M. tanarius*, *H. giganteus*, *S. indica*, and *E. atoto* extracts against *M. catarrhalis* ATCC 25238 (MIC = 12.5–25 µg/mL). The detected antibacterial effects of *M. tanarius* and *H. giganteus* can support their application for different skin disorders, fever, cough, and eye diseases. The antifungal screening proved the activity of all plants against *C. parapsilosis* ATCC 22019 and/or *N. glabrata* ATCC 2001 with MICs of 50–500 µg/mL. The strongest activity was observed for the *M. tanarius* (MIC 50 µg/mL) and *M. mollissimus* (MIC 100 µg/mL) ethyl acetate extracts.

The antitumour activities of 28 plant extracts were screened in drug-sensitive Colo 205 and drug-resistant Colo 320 colon adenocarcinoma cells. Remarkable antiproliferative activities (IC_50_ = 0.23–2.60 µg/mL) were displayed by the lipophilic extracts of *H. giganteus*, *M. rufidulus*, *S. indica*, and *E. atoto*, indicating that these extracts can serve as excellent sources of compounds with strong antitumour activity. Another interesting observation was the selective antiproliferative effect of the *M. tanarius* chloroform extract in Colo 320 cells, suggesting the presence of compounds with drug resistance-modifying activity in this extract. 

The *E. atoto n*-hexane extract, which exhibited strong antiproliferative activity, was subjected to a detailed phytochemical investigation, and five compounds, 24-methylene-cycloartan-3*β*-ol (**1**), jolkinolide E (**2**), tetra-*tert*-butyl-diphenyl ether (**3**), *α*-tocopherol (**4**), and *β*-sitosterol (**5**) were isolated. Compounds **1**–**4** were detected in this species for the first time in this study. Antitumour testing of 24-methylene-cycloartan-3*β*-ol (**1**) revealed its antiproliferative effect for the first time against HTB-26 human breast (IC_50_ = 24.83 µM) and Colo 205 human colon adenocarcinoma cells (IC_50_ 30.68 µM) without tumour cell selectivity. It is most probable that the compounds of the *E. atoto n*-hexane extract responsible for its strong antitumour activity could only partially be identified in the present experiment. Therefore, a bioassay-guided isolation procedure starting from a larger amount of extract will be needed to fully identify the active ingredients.

## Figures and Tables

**Table 1:** The use of the selected Euphorbiaceae species by category.

Treatment Categories	No. Indications or Symptoms
Skin	17
General symptoms	10
Digestive system	9
Musculoskeletal system	6
Genital system	3
Pregnancy and childbearing	3
Eye	2
Neurological system	2
Respiratory system	1
Blood, blood-forming organs and immune system	1
Endocrine, metabolic and nutritional system	1
Urinary system	1
**Total**	**56**

**Table 3:** MICs in µg/mL of the selected fractions of Euphorbiaceae species against bacteria and fungi using the broth microdilution method.

Species	Fr. ^a^	Bacteria					Fungi	
		*S. aureus* ATCC 29213	MRSA ATCC 43300	*S. epidermidis* ATCC 12228	*B. subtilis* ATCC 6633	*M. catarrhalis* ATCC 25238	*C. parapsilosis* ATCC 22019	*N. glabrata* ATCC 2001
*Euphorbia atoto*	E					12.5		250
	MW						>500	500
*Euphorbia* *hypericifolia*	C							>500
	E						500	250
	MW							500
*Homalanthus giganteus*	E	250	250	25		12.5	>500	200
	MW							500
*Macaranga tanarius*	C			15.6			500	
	E	500	250	100		12.5		50
*Mallotus* *mollissimus*	C							500
	E							100
*Mallotus* *rufidulus*	C				25			250
	E							250
*Shirakiopsis* *indica*	H							>500
	C							>500
	E						500	250
	MW					25	500	250
Ciprofloxacin		0.01	0.21	0.1	0.02	0.02		
Ampicillin		0.26	4.14	1.04	0.01	0.003		
Nystatin							0.3125	0.3125
DMSO (%)	>2%	>2%	>2%	>2%	>2%	>2%	>2%	>2%

^a^ H = *n*-hexane, C = chloroform, E = ethyl acetate, MW = aqueous methanolic extract.

**Table 4:** Antiproliferative activities of the plant extracts in colorectal adenocarcinoma cells.

Plant Name		IC_50_ Value (µg/mL) ^a^	
	Fraction	Colo 205	Colo 320
*Euphorbia atoto*	*n*-Hex	0.24 ± 0.06	55.02 ± 0.91
	CHCl_3_	0.23 ± 0.04	53.17 ± 0.49
	EtOAc	N	N
	Aq-MeOH	-	-
*Euphorbia hypericifolia*	*n*-Hex	55.94 ± 0.64	72.05 ± 0.82
	CHCl_3_	N	36.54 ± 0.64
	EtOAc	N	N
	Aq-MeOH	N	N
*Homalanthus giganteus*	*n*-Hex	0.92 ± 0.15	31.92 ± 2.69
	CHCl_3_	14.42 ± 1.30	26.18 ± 1.77
	EtOAc	N	N
	Aq-MeOH	N	N
*Macaranga tanarius*	*n*-Hex	56.58 ± 1.14	66.57 ± 2.01
	CHCl_3_	23.02 ± 0.86	8.46 ± 0.36
	EtOAc	N	N
	Aq-MeOH	N	N
*Mallotus mollissimus*	*n*-Hex	37.29 ± 1.13	84.40 ± 1.22
	CHCl_3_	57.18 ± 0.41	62.03 ± 0.42
	EtOAc	N	N
	Aq-MeOH	N	N
*Mallotus rufidulus*	*n*-Hex	23.18 ± 2.22	57.35 ± 0.58
	CHCl_3_	0.56 ± 0.07	7.10 ± 0.60
	EtOAc	N	N
	Aq-MeOH	N	N
*Shirakiopsis indica*	*n*-Hex	2.60 ± 0.35	55.53 ± 1.54
	CHCl_3_	1.04 ± 0.01	11.93 ± 0.82
	EtOAc	N	N
	Aq-MeOH	N	N
Doxorubicin ^b^		1.45 ± 0.19	1.76 ± 0.09

^a^ IC_50_ values are means of triplicate (n = 3) ± standard deviations. N, no IC_50_ shown or the IC_50_ was above 100 µg/mL. IC_50_ of DMSO, >2% *v*/*v*. ^b^ Positive control.

**Table 5:** Antiproliferative effects of **1** (IC_50_ in µM).

Cell Strain	Compound 1		Doxorubicin		Cisplatin	
	Mean	SD	Mean	SD	Mean	SD
Colo 205	30.68	0.70	0.33	0.009	24.09	1.47
HTB-26	24.83	2.46	0.37	0.021	26.64	1.82
MCF-7	>100	-	0.60	0.045	20.61	1.26
MRC-5	15.67	0.59	0.50	0.033	2.79	0.17

**Table 6:** Scientific name, location of plant collection, and plant parts used in the experiments.

Voucher No	Scientific Name	Author Name	Place of Collection	Plant Part
DGW-07	*Euphorbia atoto*	G.Forst.	Gerupuk bay, Central Lombok	Aerial parts
DGW-08	*Euphorbia hypericifolia*	L.	Sulat, East Lombok	Aerial parts
DGW-03	*Homalanthus giganteus*	Zoll. & Moritzi	Mareje, West Lombok	Leaves
DGW-01	*Macaranga tanarius*	Müll.Arg.	Mareje, West Lombok	Leaves
DGW-02	*Mallotus mollissimus*	(Geiseler) Airy Shaw	Mareje, West Lombok	Leaves
DGW-04	*Mallotus rufidulus*	Müll.Arg.	Mareje, West Lombok	Leaves
DGW-05	*Shirakiopsis indica*	(Willd.) Esser	Pujut, Central Lombok	Leaves

**Table 7:** Yields of plant extracts and fractions.

Plants (Dry Weight)	Part	MeOH (L)	Yield g (%)				
			MeOH ext.	*n*-Hex fr.	CHCl_3_ fr.	EtOAc fr.	Aq-MeOH fr.
*M. tanarius* (60.06 g)	Leaves	2.4	15.45	1.36	3.60	2.38 g	6.16 g
			(25.72)	(8.80)	(23.30)	(15.40)	(39.87)
*M. mollissimus* (69.15 g)	Leaves	3.0	17.75	4.27	0.72	0.07	11.60
			(25.67)	(24.06)	(4.06)	(0.39)	(65.35)
*H. giganteus* (116.4 g)	Leaves	3.0	51.39	6.37	1.84	2.22	19.7
			(44.14)	(12.39)	(3.58)	(4.32)	(38.33)
*M. rufidulus* (54.12 g)	Leaves	2.8	21.15	2.65	0.46	0.25	6.29
			(39.08)	(12.53)	(2.17)	(1.18)	(29.74)
*S. indica* (117.61 g)	Leaves	4.0	77.98	12.70	0.56	8.00	31.62
			(66.30)	(16.28)	(0.72)	(10.26)	(40.54)
*E. atoto* (234.70 g)	Aerial parts	4.1	83.55	8.12	0.78	0.08	47.80
			(35.59)	(9.71)	(0.93)	(0.09)	(57.21)
*E. hypericifolia* (14.42 g)	Aerial parts	0.8	3.34	0.32	0.20	1.06	1.10
			(24.20)	(9.58)	(5.99)	(31.73)	(32.93)

## Data Availability

Data are contained within the article and supplementary materials.

## Supplementary Materials

The following supporting information can be downloaded at: https://www.mdpi.com/article/10.3390/plants12223836/s1, Figures S1–S12, NMR spectra of compounds **1**–**4**, and Table S1: Antimicrobial activities of the fractions at a concentration of 50 µg/mL by the agar disk diffusion method.
