# Supplementary material for: Indonesian Euphorbiaceae: Ethnobotanical Survey, In Vitro Antibacterial, Antitumour Screening and Phytochemical Analysis of Euphorbia atoto
Source: Plants (Basel). 2023 Nov 13;12(22):3836. doi: 10.3390/plants12223836 (PMC10675575; doi:10.3390/plants12223836)
Supplement: Supplementary file 1 [file plants-12-03836-s001.zip › plants-2692639-supplementary.pdf]

SUPPORTING INFORMATION

Indonesian Euphorbiaceae: Ethnobotanical Survey, In Vitro Antibacterial, Antitumour Screening and Phytochemical Analysis of *Euphorbia atoto*

Dyke Gita Wirasisya [1,2], Annamária Kincses [1], Lívia Vidács [1], Nikoletta Szemerédi [3], Gabriella Spengler [3], Anita Barta [1], I Gde Mertha [4] and Judit Hohmann [1,5,*]

[1] Institute of Pharmacognosy, University of Szeged, H-6720 Szeged, Hungary; dykegita_w@unram.ac.id (D.G.W.); kincses.annamaria@szte.hu (A.K.); vidacs.livia@gmail.com (L.V.); bartaanita96@gmail.com (A.B.)

[2] Department of Pharmacy, Faculty of Medicine, University of Mataram, Mataram 83126, Indonesia

[3] Department of Medical Microbiology, Albert Szent-Györgyi Health Center and Albert Szent-Györgyi Medical School, University of Szeged, H-6725 Szeged, Hungary; szemeredi.nikoletta@med.u-szeged.hu (N.S.); spengler.gabriella@med.u-szeged.hu (G.S.)

[4] Department of Biology Education, Faculty of Teacher Training and Education, University of Mataram, Mataram 83126, Indonesia; gdemerth19@gmail.com

[5] ELKH-USZ Biologically Active Natural Products Research Group, University of Szeged, H-6720 Szeged, Hungary

* Correspondence: hohmann.judit@szte.hu; Tel.: +36-62-546453

CONTENT

Figure S1. ^{1}H NMR spectrum of compound **1** (500 MHz, $CDCl_3$) 2

Figure S2. ^{13}C NMR JMOD spectrum of compound **1** (125 MHz, $CDCl_3$) 2

Figure S3. ^{1}H NMR spectrum of compound **2** (500 MHz, $CDCl_3$) 3

Figure S4. ^{13}C NMR JMOD spectrum of compound **2** (125 MHz, $CDCl_3$) 3

Figure S5. ^{1}H-^{1}H COSY spectrum of compound **2** (500 MHz, $CDCl_3$) 4

Figure S6. HSQC spectrum of compound **2** 4

Figure S7. HMBC spectrum of compound **2** 5

Figure S8. NOESY spectrum of compound **2** 5

Figure S9. ^{1}H NMR spectrum of compound **3** (500 MHz, $CDCl_3$) 6

Figure S10. ^{13}C NMR JMOD spectrum of compound **3** (125 MHz, $CDCl_3$) 6

Figure S11. ^{1}H NMR spectrum of compound **4** ($CDCl_3$, 500 MHz) 7

Figure S12. ^{13}C NMR JMOD spectrum of compound **4** ($CDCl_3$, 125 MHz) 7

Table S1. Antimicrobial activities of the fractions at a concentration of 50 µg/mL by the agar disk diffusion method 8

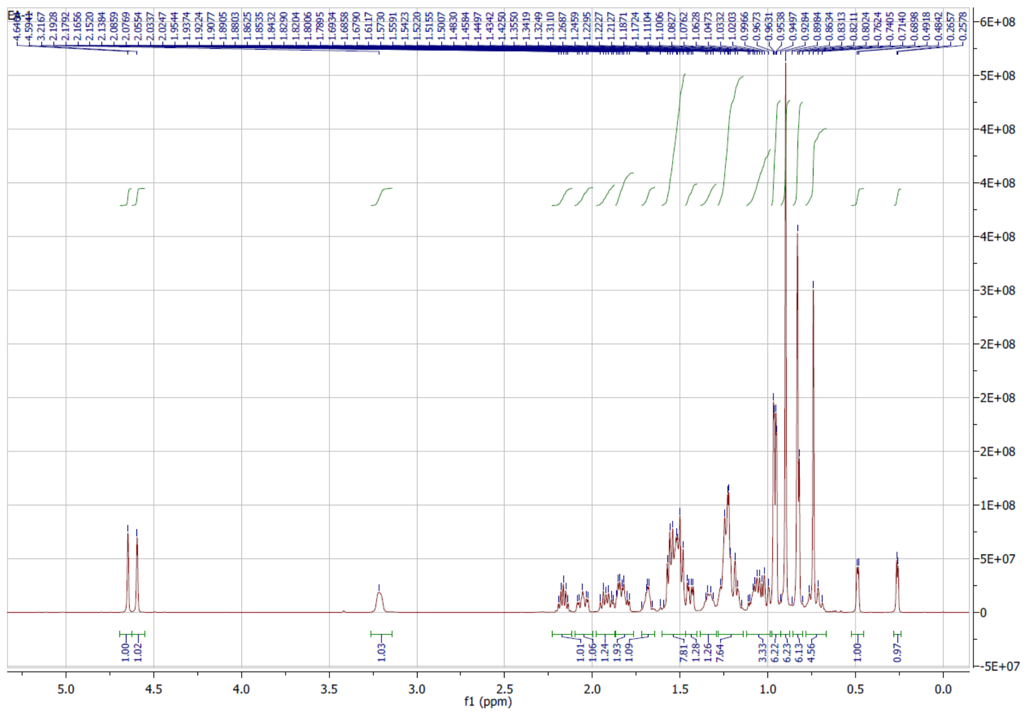

Figure S1. ^{1}H NMR spectrum of compound **1** (500 MHz, $CDCl_3$)

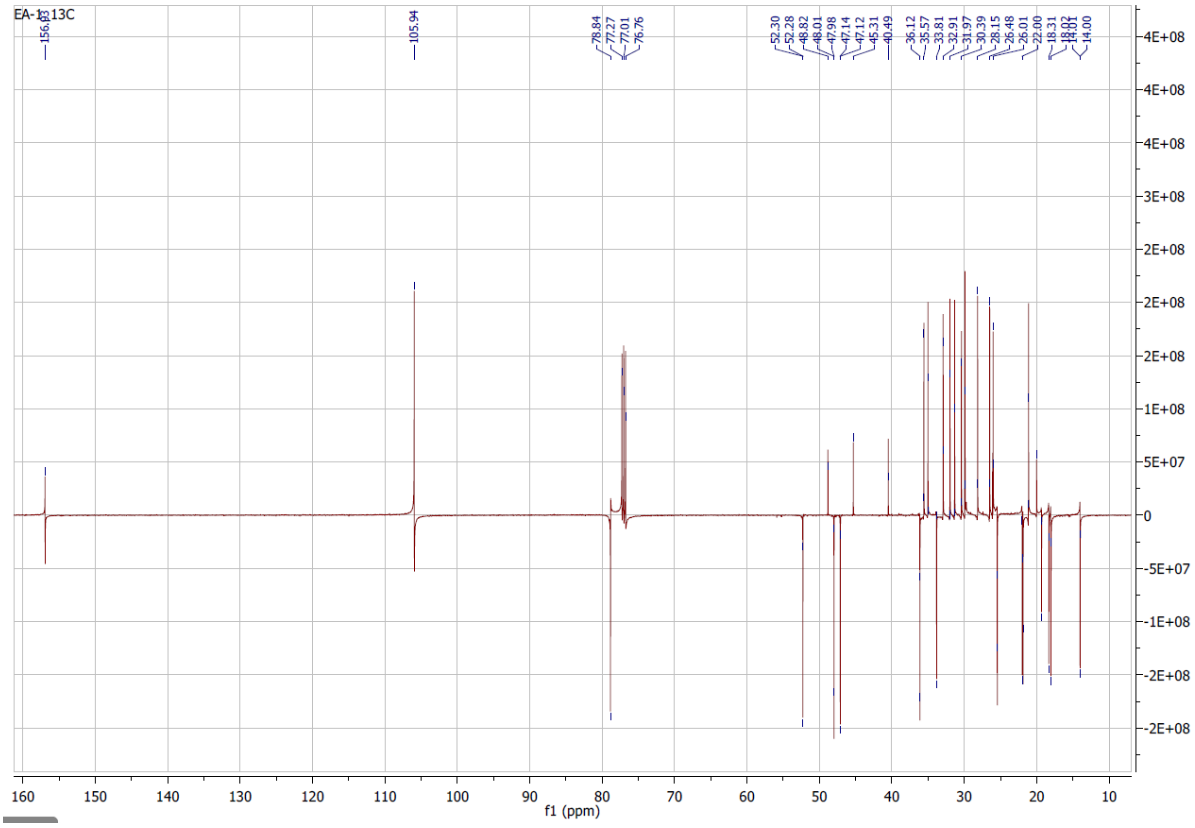

Figure S2. ^{13}C NMR JMOD spectrum of compound **1** (125 MHz, $CDCl_3$)

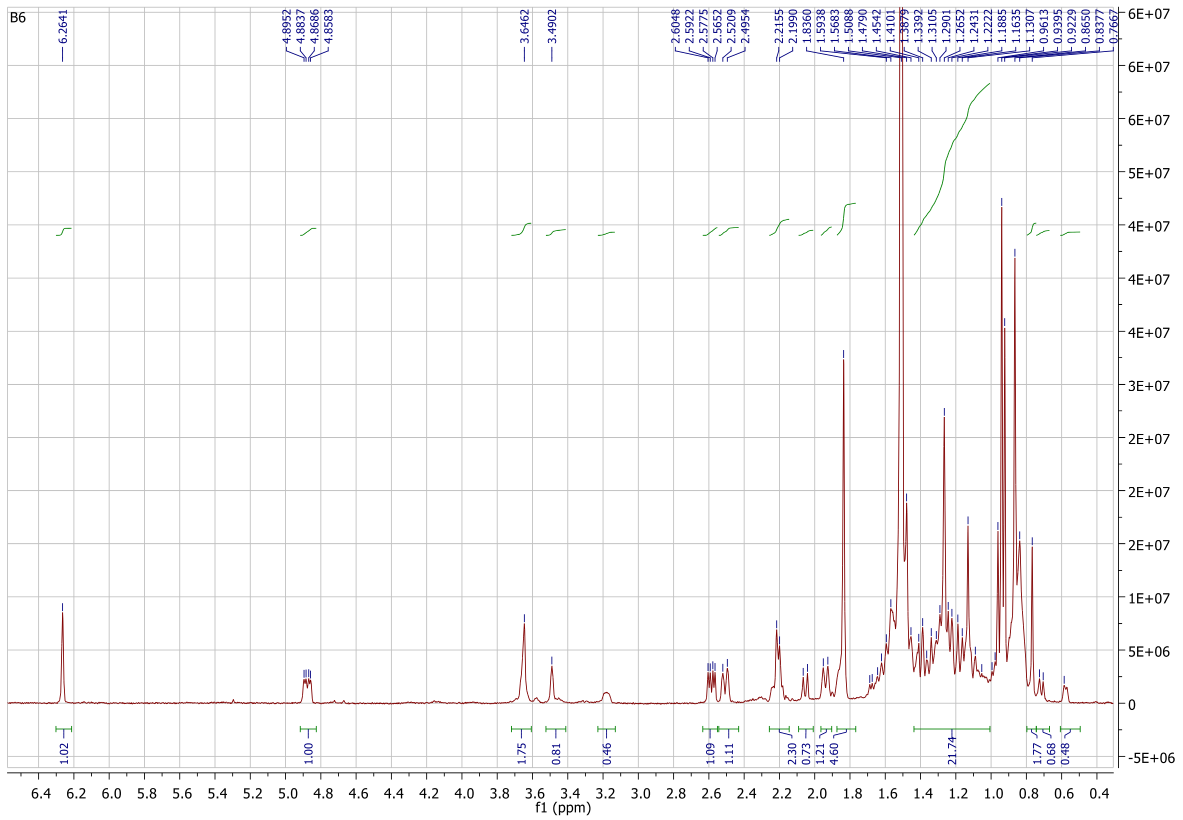

Figure S3. ^{1}H NMR spectrum of compound **2** (500 MHz, $CDCl_3$)

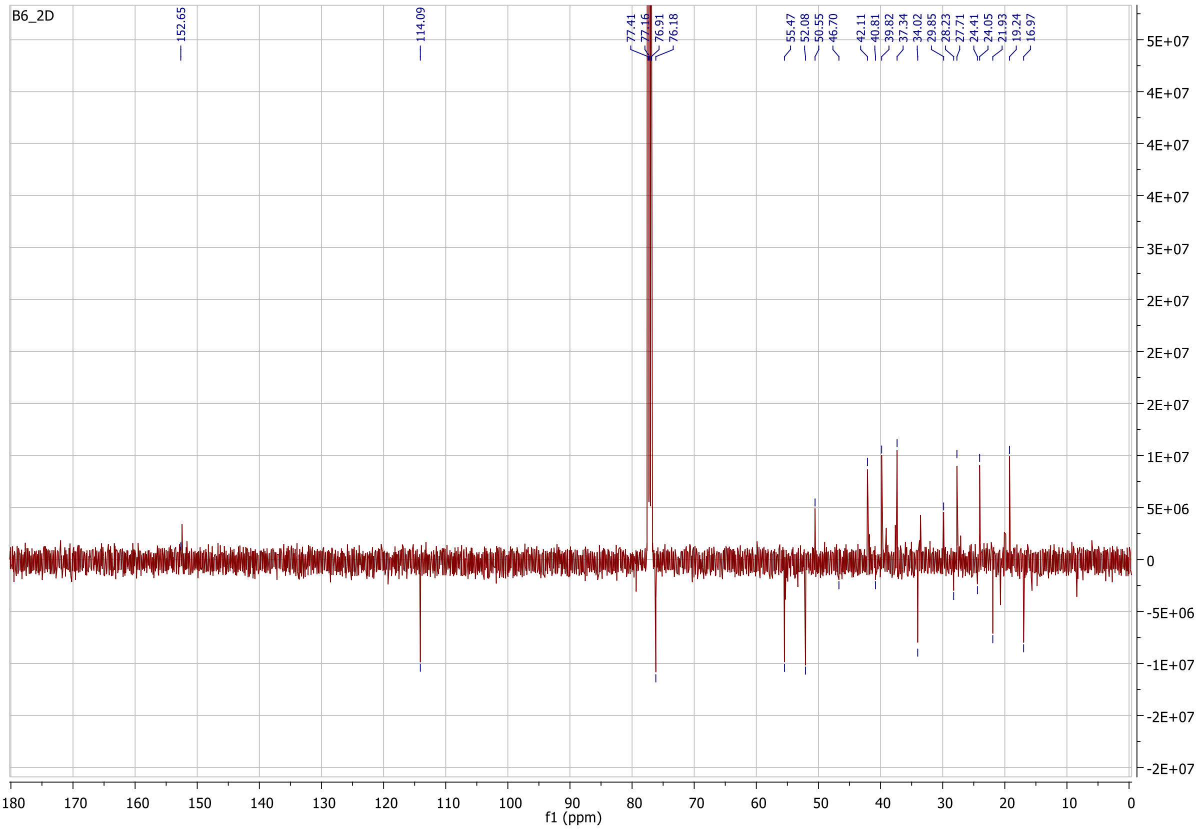

Figure S4. ^{13}C NMR JMOD spectrum of compound **2** (125 MHz, $CDCl_3$)

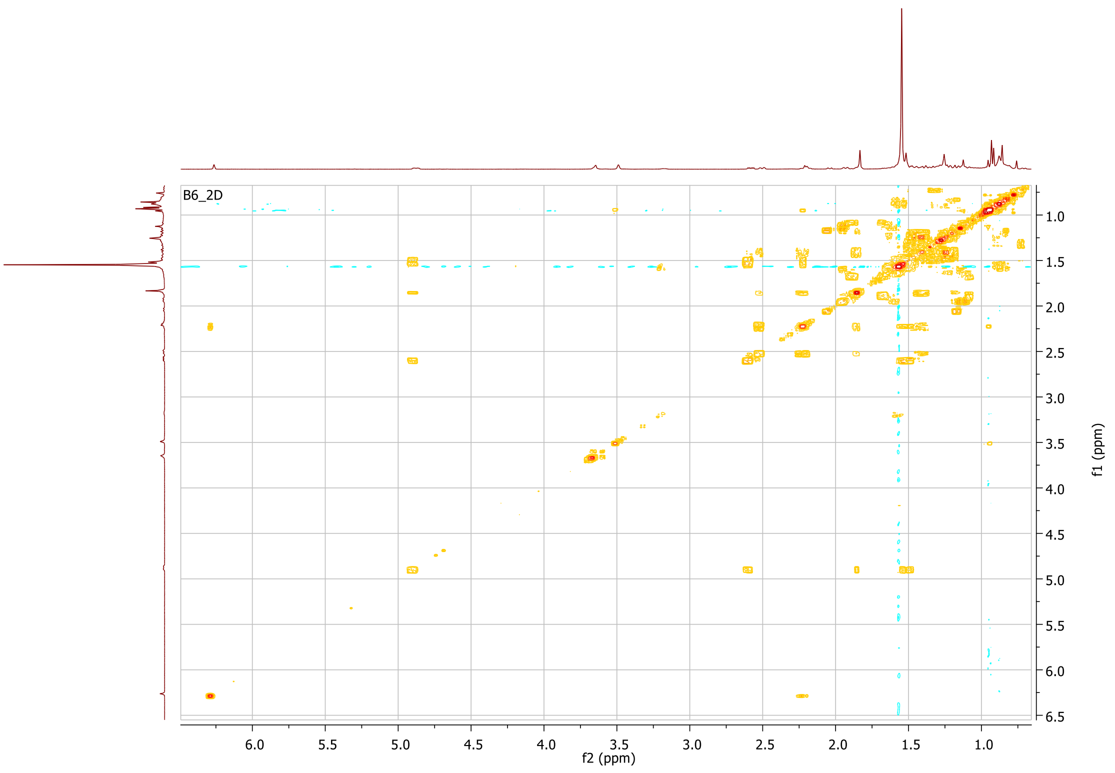

Figure S5. ^1H-^1H COSY spectrum of compound **2** (500 MHz, $CDCl_3$)

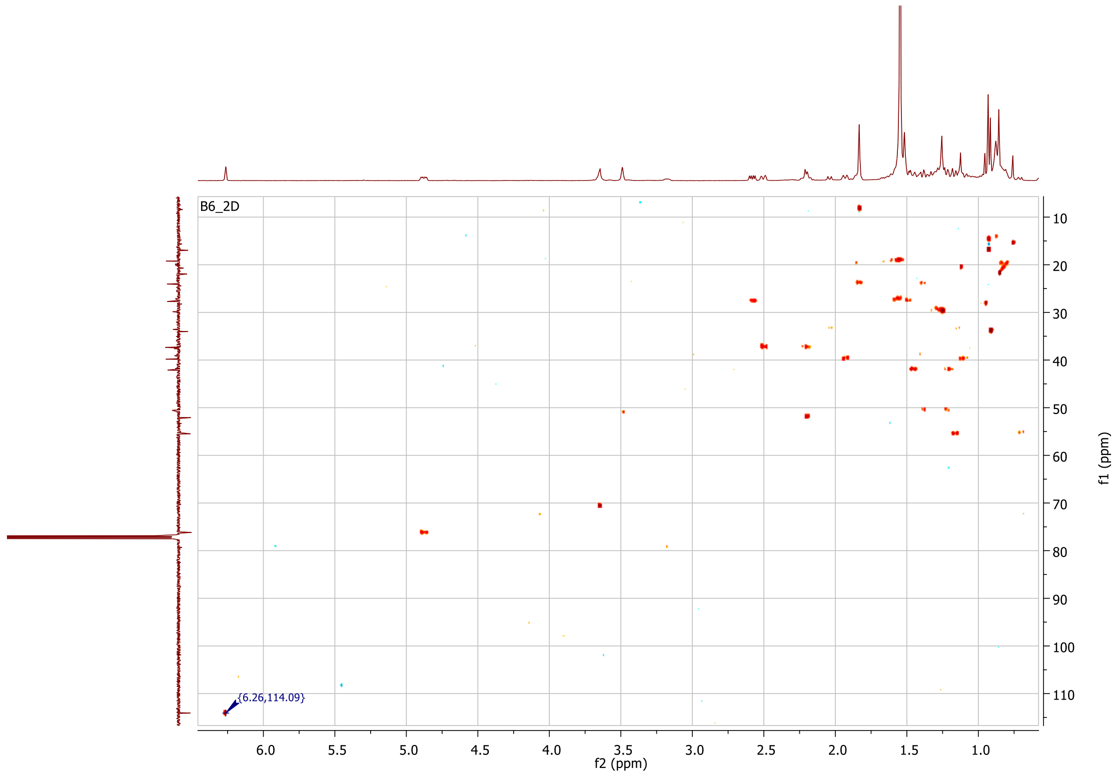

Figure S6. HSQC spectrum of compound **2**

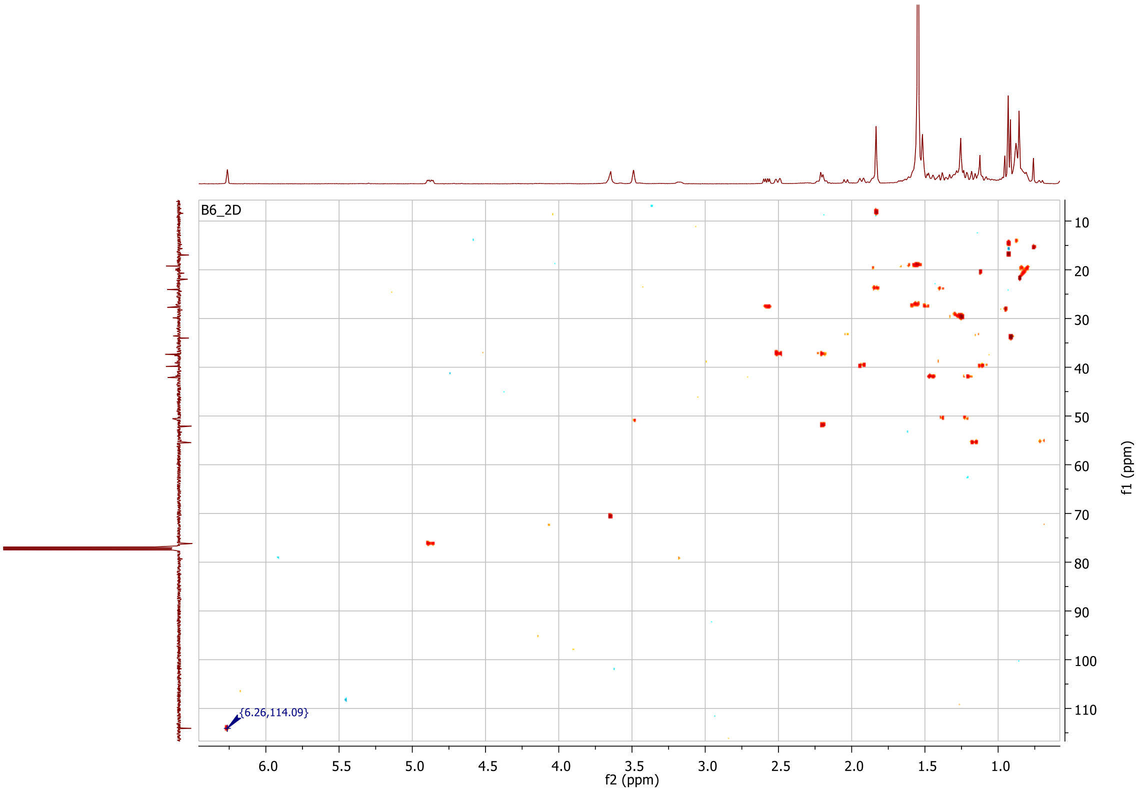

Figure S7. HMBC spectrum of compound **2**

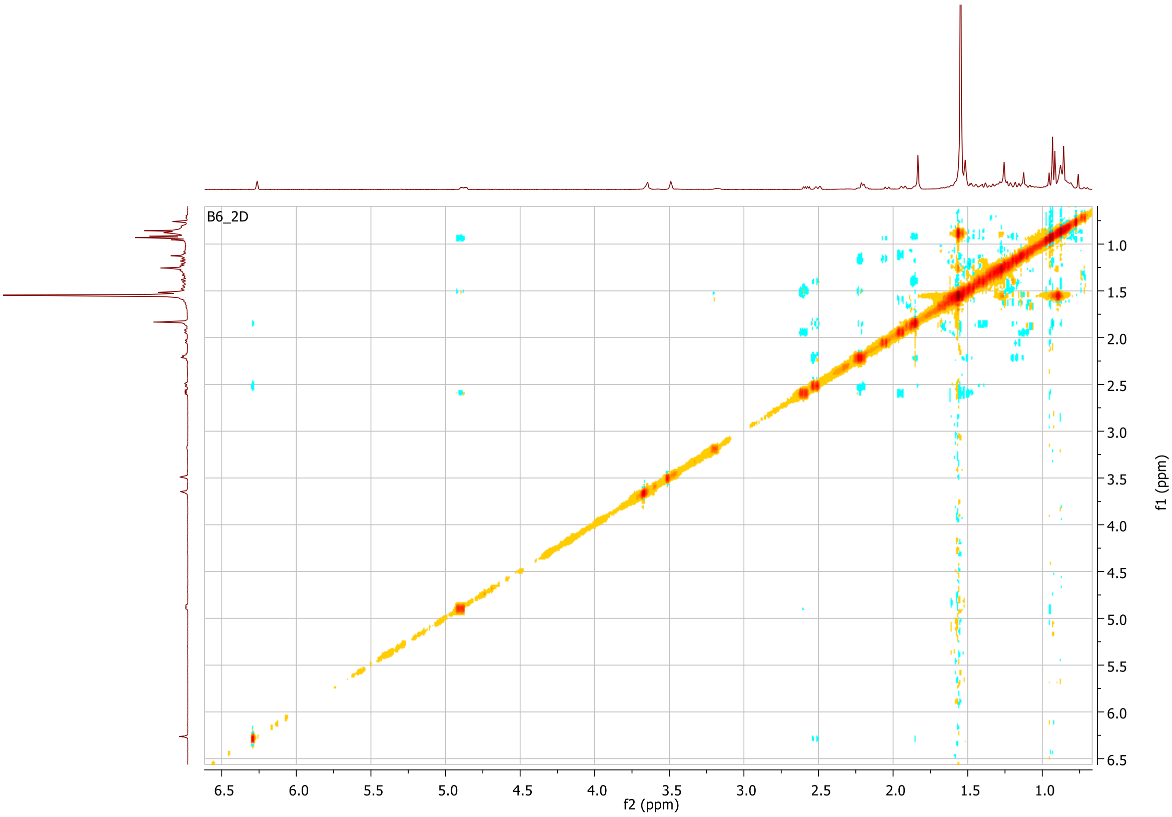

Figure S8. NOESY spectrum of compound **2**

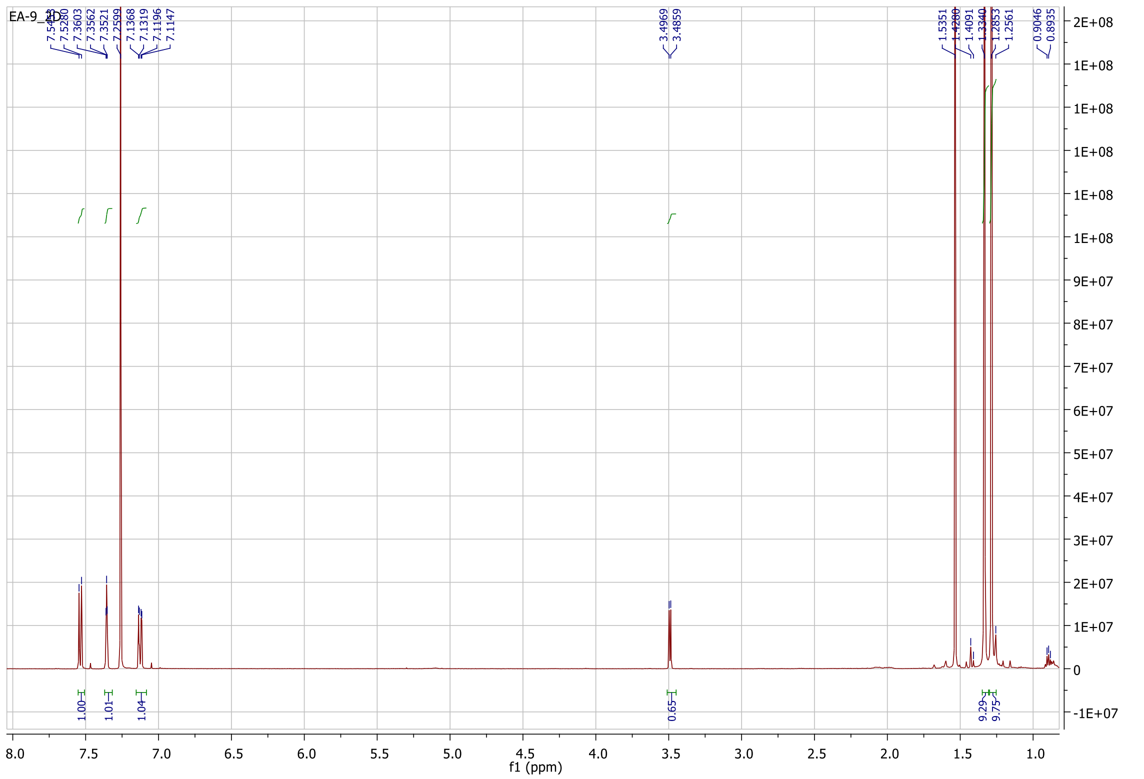

Figure S9. ^{1}H NMR spectrum of compound **3** (500 MHz, $CDCl_3$)

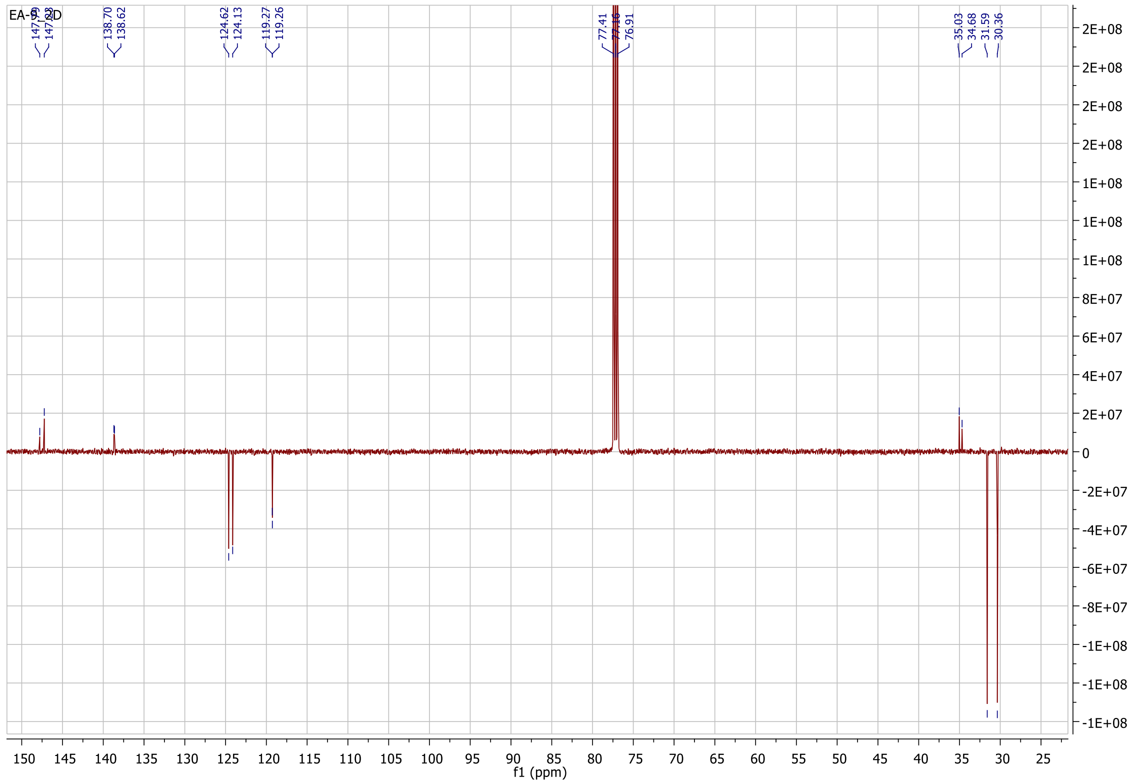

Figure S10. ^{13}C NMR JMOD spectrum of compound **3** (125 MHz, $CDCl_3$)

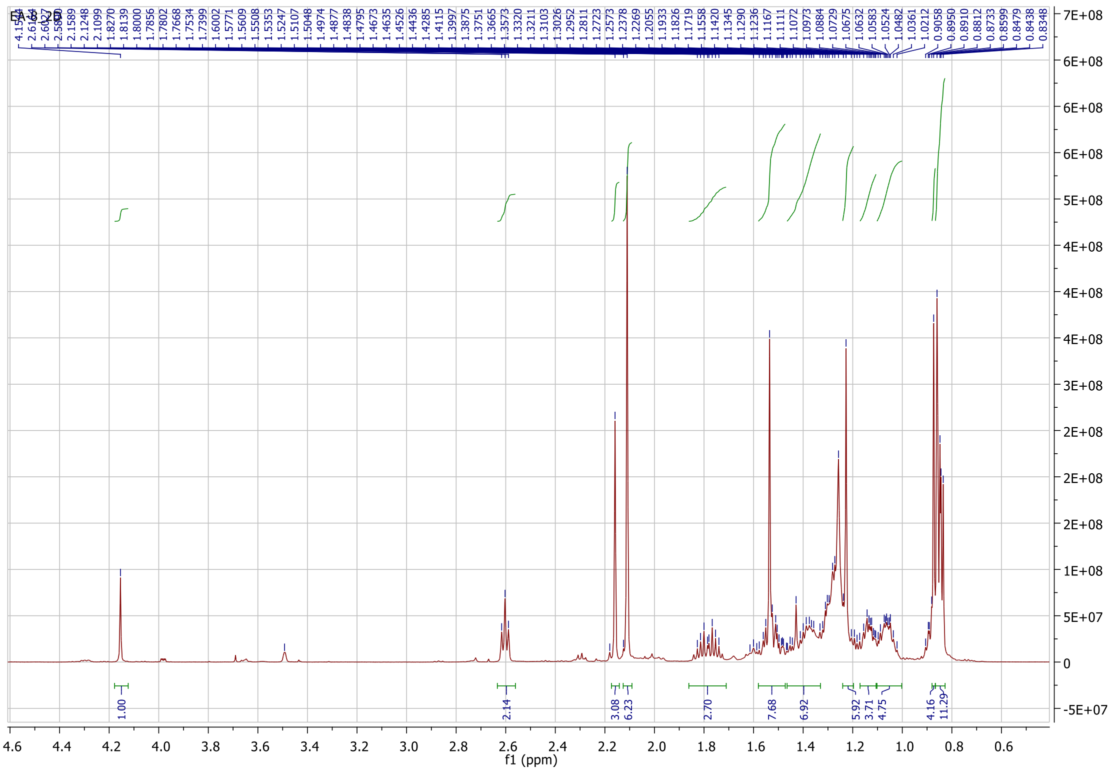

Figure S11. ^{1}H NMR spectrum of compound **4** ($CDCl_3$, 500 MHz)

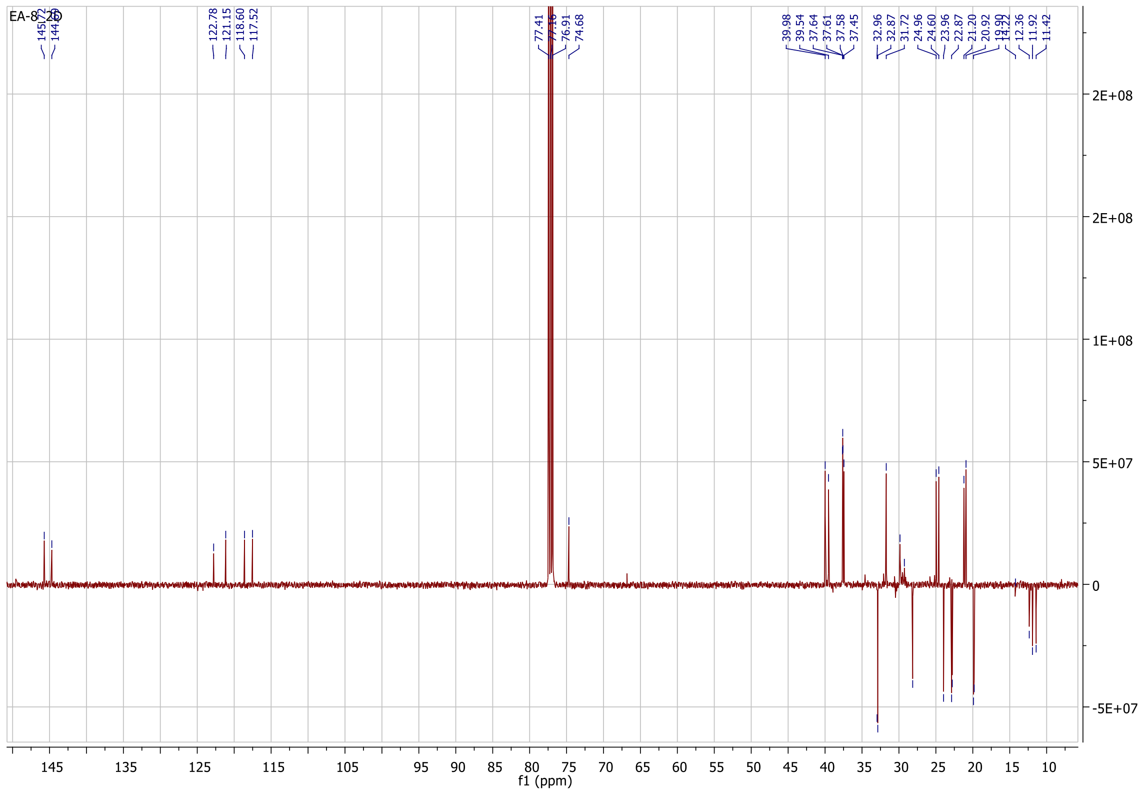

Figure S12. ^{13}C NMR JMOD spectrum of compound **4** ($CDCl_3$, 125 MHz)

Table S1. Antimicrobial activities of the fractions at a concentration of 50 µg/mL by the agar disk diffusion method.

		**Zone of inhibition (mm)**[a]														
		**Gram-positive bacteria**							**Gram-negative bacteria**				**Fungi**			
**Plant**	**Extract**	***S. aureus* ATCC 29213**	***MRSA* ATCC *43300***	***S. epidermidis* ATCC 12228**	***E. faecalis* ATCC 29212**	***S. pyogenes* ATCC 19615**	***S. agalactiae* ATCC 13813**	***B. subtilis* ATCC 6633**	***E. coli* ATCC 35218**	***E. coli* K-12 AG-100**	***P. aeruginosa* ATCC 27853**	***M. catarrhalis* ATCC 25238**	***C. parapsilosis* ATCC 22019**	***N. glabrata* ATCC 2001**	***C. tropicalis* ATCC 750**	***C. albicans* ATCC 10231**
Macaranga tanarius	H	9±0.0	9±0.0	11±0.0	N	N	8±0.0	10±0.0	N	N	N	9±0.6	N	N	N	N
	C	13±0.0	13±0.2	**16±0.0**	11±0.0	9±0.0	8±0.4	13±0.0	N	7±0.0	N	10±0.0	N	N	7±0.0	8±0.0
	E	**15±0.0**	**15±0.0**	**15±0.0**	N	5.3±4.6	N	7±0.0	N	N	N	**18±0.3**	**20±0.0**	**17±0.0**	7±0.0	8±0.6
	MW	10±0.0	N	13±0.0	N	N	N	N	N	N	N	13±0.0	N	N	N	N
Mallotus mollisimus	H	11±0.0	10±0.0	N	N	7±0.0	7±0.0	12±0.0	N	N	N	8±0.0	7±0.0	7±0.0	7±0.0	7±0.0
	C	8±0.0	9±0.1	N	N	10±0.0	7±0.0	9±0.0	N	N	N	8±0.0	N	**15±0.1**	N	N
	E	7±0.0	N	N	N	9±0.0	N	N	N	N	N	N	N	**20±0.0**	N	N
	MW	9±0.0	N	9±0.0	N	N	N	N	N	N	N	8±0.1	N	N	N	N
Homalanthus giganteus	H	N	N	N	N	N	N	N	N	N	N	N	N	14±0.0	N	N
	C	N	N	N	N	N	N	7±0.0	N	N	N	7±0.0	N	13±0.0	N	N
	E	**15±0.0**	**15±0.4**	**15±0.0**	8±0.0	7±0.1	N	8±0.0	N	N	N	**16±0.0**	**18±0.0**	**25±0.0**	N	N
	MW	N	N	9±0.0	N	N	N	N	N	N	N	9±0.0	N	**18±0.0**	N	N
Mallotus rufidulus	H	10±0.0	13±0.0	8±0.0	N	N	N	11±0.0	N	N	N	9±0.0	N	N	N	N
	C	13±0.0	12.6±1.2	10±0.3	N	10±0.0	N	**16±0.0**	N	N	N	10±0.0	N	N	N	N
	E	9±0.0	10±0.0	10±0.0	N	N	N	8±0.0	N	N	N	10±0.0	N	**18±0.0**	8±0.0	N
	MW	N	N	N	N	N	N	N	N	N	N	N	N	N	N	N

Shirakiopsis indica	H	N	N	7±0.0	N	N	N	N	N	N	N	7±0.0	N	**22±0.0**	N	8±0.0
	C	N	N	N	N	8±0.0	7±0.0	8±0.0	N	N	N	10±0.0	N	**30±0.2**	N	N
	E	N	N	N	N	N	N	N	N	N	N	12±0.0	**23±0.0**	**33±0.0**	N	N
	MW	12±0.0	12±0.2	N	N	N	N	N	N	N	N	**15±0.0**	**17±0.0**	**22±0.0**	N	N
Euphorbia atoto	H	N	N	N	N	N	N	N	N	N	N	N	N	12±0.0	N	N
	C	8±0.0	7±0.0	N	N	N	N	7±0.0	N	N	N	N	N	N	N	N
	E	8±0.0	8±0.0	7±0.6	7±0.0	N	7±0.0	9±0.3	N	7±0.0	N	10±0.0	N	**20±0.0**	7±0.0	N
	MW	12±0.0	12±0.0	13±0.0	N	7±0.0	N	N	N	N	N	11±0.0	**19±0.0**	**20±0.0**	N	N
Euphorbia hypericifolia	H	N	N	N	N	N	N	N	N	N	N	N	N	10.6±1.2	N	N
	C	7±0.4	N	N	N	7±0.0	N	7±0.0	N	N	N	N	N	**19±0.0**	N	N
	E	10±0.2	12±0.0	9±0.3	N	N	7±0.0	N	N	N	N	13±0.0	**24±0.0**	**28±0.0**	N	10±0.0
	MW	11±0.0	N	14±0.0	N	N	N	N	N	N	N	10±0.0	N	**26±0.0**	N	N
Ciprofloxacin (5 µg)		30±0.0	27±0.0	34±0.0	20±0.0	18±0.0	17±0.0	28±0.0	30±0.0	30±0.0	28±0.0	30	N/A	N/A	N/A	N/A
Ampicillin (10 µg)		18±0.0	10±0.0	16±0.0	26±0.0	30±0.0	15±0.0	22±0.0	20±0.0	24±0.0	N	34	N/A	N/A	N/A	N/A
Nystatin (5 µg)		N/A	N/A	N/A	N/A	N/A	N/A	N/A	N/A	N/A	N/A	N/A	20±0.2	21±0.0	24±0.0	20±0.0

[a] Values are means of triplicate (n = 3) ± standard deviations. N, no inhibition shown. N/A, not applicable.
H = *n*-hexane, C = chloroform, E = ethyl acetate, MW aqueous methanolic extract.
